# GnRH receptor gene mutations in adolescents and young adults presenting with signs of partial gonadotropin deficiency

**DOI:** 10.1371/journal.pone.0188750

**Published:** 2017-11-28

**Authors:** Johanna Hietamäki, Matti Hero, Elina Holopainen, Johanna Känsäkoski, Kirsi Vaaralahti, Anna-Pauliina Iivonen, Päivi J. Miettinen, Taneli Raivio

**Affiliations:** 1 Pediatric Research Center, Children’s Hospital, University of Helsinki and Helsinki University Hospital, Helsinki, Finland; 2 Department of Physiology, Faculty of Medicine, University of Helsinki, Helsinki, Finland; 3 Department of Obstetrics and Gynecology, University of Helsinki and Helsinki University Hospital, Helsinki, Finland; 4 Research Programs Unit, Molecular Neurology, and Biomedicum Stem Cell Center, University of Helsinki, Helsinki, Finland; John Hopkins University School of Medicine, UNITED STATES

## Abstract

Biallelic, partial loss-of-function mutations in *GNRHR* cause a wide spectrum of reproductive phenotypes from constitutional delay of growth and puberty to complete congenital hypogonadotropic hypogonadism. We studied the frequency of *GNRHR*, *FGFR1*, *TAC3*, and *TACR3* mutations in nine adolescent and young adult females with clinical cues consistent with partial gonadotropin deficiency (stalled puberty, unexplained secondary amenorrhea), and describe phenotypic features and molecular genetic findings of monozygotic twin brothers with stalled puberty.

Two girls out of nine (22%, 95%CI 6–55%) carried biallelic mutations in *GNRHR*. The girl with compound heterozygous c.317A>G p.(Gln106Arg) and c.924_926delCTT p.(Phe309del) *GNRHR* mutations displayed incomplete puberty and clinical signs of hypoestrogenism. The patient carrying a homozygous c.785G>A p.(Arg262Gln) mutation presented with signs of hypoestrogenism and unexplained secondary amenorrhea. None of the patients exhibited mutations in *FGFR1*, *TAC3*, or *TACR3*. The twin brothers, compound heterozygous for *GNRHR* mutations c.317A>G p.(Gln106Arg) and c.785G>A p.(Arg262Gln), presented with stalled puberty and were discordant for weight, and the heavier of them had lower testosterone levels.

These results suggest that genetic testing of the *GNRHR* gene should be offered to adolescent females with low-normal gonadotropins and unexplained stalled puberty or menstrual dysfunction. In male patients with partial gonadotropin deficiency, excess adipose tissue may suppress hypothalamic-pituitary-gonadal axis.

## Introduction

During the last four years, the scientific community has witnessed significant advances in the identification of high-impact genetic factors that determine the timing of puberty. Especially, the discovery of paternally inherited mutations in *MKRN3* as a cause of precocious puberty in both sexes, and mutations in *IGSF10* in a significant proportion of patients with delayed puberty are findings that direct diagnostics and enable early counselling and patient assurance [1,2]. In both scenarios, while the initial timing of the onset and/or pace of pubertal progression deviates from the population mean, the endogenous puberty *per se* will be completed and the reproductive capacity is intact. This is not the case in patients with congenital hypogonadotropic hypogonadism (CHH), a rare disease that refers to deficient central regulation of the gonadal function, which typically manifests as absent or delayed puberty [3]. The diagnosis of CHH is often challenging especially in patients with partial forms, due to the difficulty in differentiating CHH from the constitutional delay of growth and puberty (CDGP) or functional hypothalamic amenorrhea [4–7].

We have previously investigated the frequency of *GNRHR* mutations in a well-phenotyped cohort of patients with CDGP [8], but did not find any patients with biallelic mutations. Similar findings were recently reported from Brazil [9]. We therefore anticipated that *GNRHR* mutations leading to partial loss-of-function, such as p.(Arg262Gln) and p.(Gln106Arg) [10,11] should be sought for in patients with clinical and biochemical signs of mild gonadotropin deficiency, such as problems in the progression of puberty and/or maintenance of the hypothalamic-pituitary-gonadal axis. In addition, the seminal paper by Caronia *et al*. [4] suggested that mutations in genes implicated in CHH could explain a significant proportion of patients presenting with hypothalamic amenorrhea. Indeed, the clinical presentation of CHH in females is notably heterogeneous: 12% of patients had experienced isolated menses, and up to 50% presented with some degree of breast development [9,12]. In males, biallelic partial loss-of-function mutations in *GNRHR* are known to cause a wide spectrum of reproductive phenotypes ranging from delayed puberty and CHH, to reversal and relapse of CHH [11,13,14], although the role of environmental factors in modifying the phenotype has been difficult to address.

Knowing the previously described phenotypic variability of patients with *GNRHR* mutations, we hypothesized that a significant proportion of adolescent and young adult patients presenting with signs of partial gonadotropin deficiency (manifesting as stalled puberty or unexplained secondary amenorrhea) harbor biallelic *GNRHR* mutations. In addition, we describe monozygotic twins with biallelic *GNRHR* mutations who were discordant for weight and displayed fascinating temporal variation in their phenotype.

## Patients and methods

Adolescent and young adult female patients (mean age at recruitment 21.2±1.85 years (range, 18.1–25.6 years)) visiting the Helsinki University Hospital Gynaecology outpatient clinic (clinician E. H.) between Sep 2015 and Mar 2016 (6 patients) and Dec 2016 to Jan 2017 (3 patients) with findings suggesting partial gonadotropin deficiency, manifesting as stalled puberty (*i*.*e*. low or normal gonadotropin levels associated with spontaneous onset of breast development with lack of progression) or unexplained secondary amenorrhea, were asked to participate in the study. Patients with factors favoring functional hypothalamic amenorrhea (HA), *i*.*e*. weight fluctuation and excessive exercise, were included only, if the experienced recruiting clinician did not find these factors to exclusively support the diagnosis of HA. Eleven patients were asked to attend the study, of whom two refused to participate. Of the nine patients enrolled, seven had primary amenorrhea and at least partial spontaneous breast development, and two patients had secondary amenorrhea, and subsequent hormone replacement therapy (HRT) induced menstrual bleeding. Three patients had a positive self-reported family history of delayed puberty.

In addition, two boys referred to the pediatric endocrine outpatient clinic for incomplete puberty were enrolled. These patients were monochorionic, diamniotic twins born at gestation week 34+5 from the mother’s first pregnancy. The pregnancy had been spontaneous and without complications, except for a pathologic oral glucose tolerance test. Amniocentesis at gestation week 16+3 demonstrated normal 46,XY karyotype for both fetuses. The twins were born with similar birth size: birth measures were 47.0 cm/ 2430 g (Twin A) and 47.5 cm/ 2525 g (Twin B). The pubertal development of the mother had been normal, with menarche occurring at 13 years of age; similarly, the father’s pubertal progression had been normal. There was no known family history of infertility or anosmia.

### Pubertal status and biochemical testing

The clinical features of the patients were assessed during the investigations of stalled puberty in the tertiary care center, and their medical histories were obtained from the patient record. Pubertal stage was assessed according to Tanner [15] and, in boys, the testes were measured to the nearest mm with a ruler, and testicular volume (TV) was calculated by using the formula TV = length x width^2^ x 0.52 [16]. Bone age was assessed by using the BoneXpert software [17].

Serum LH and FSH concentrations were measured using an electrochemiluminescence immunoassay (Roche Diagnostics, USA). Serum testosterone concentrations were measured using mass spectrometry (AB Sciex, Foster City, California, USA), serum estradiol concentrations either using immunochemical assays (AutoDelfia, Perkin-Elmer, USA, or Immulite 2000, Siemens, Germany) or mass spectrometry (Siemens Healthcare Diagnostics, Germany), inhibin-B levels using ELISA (Beckman Coulter, Inc. USA), and AMH levels using immunoassay (Immunotech, Beckman Coulter Ltd. (A79765)). The normal values for estradiol in premenopausal females (starting from Tanner stage IV) are 15–350 pg/mL* 3.767 (= 0.056 nM-1.32 nM) as they depend on the phase of menstrual cycle [18]. In the GnRH stimulation test, a 100 μg rapid bolus of GnRH (Relefact, Hoechst, Frankfurt am Main, Germany) was administered intravenously. Serum FSH levels were measured at 0 (immediately before the administration of GnRH), 30, 60, and 90 minutes, and serum LH levels at 0, 20, 30, and 60 minutes, respectively. Female patients had been off estrogen therapy for at least one month before biochemical testing. Ultrasound scans of the reproductive organs were performed by an experienced gynecologist at each visit.

### Molecular genetic analyses

Genomic DNA from saliva samples of the subjects and their relatives was extracted according to manufacturer’s instructions (Oragene DNA-kit OG-500, DNA Genotek Inc., Ottawa, Ontario, Canada). The coding exons and exon-intron boundaries of *GNRHR*, *FGFR1*, *TAC3*, and *TACR3*, were PCR-amplified, the PCR products were purified with Illustra ExoProStar treatment (GE Healthcare, Chicago, Illinois, USA), and bi-directionally sequenced using the ABI BigDyeTerminator Cycle Sequencing Kit (v3.1) and ABI Prism 3730xl DNA Analyzer automated sequencer (Applied Biosystems, Thermo Fisher Scientific, Waltham, Massachusetts, USA). The sequences were aligned and read with Sequencher 5.0 software (Gene Codes Corporation, Ann Arbor, Michigan, USA). All primer sequences are listed in S1 Table. The PCR conditions are available upon request. Monozygosity of the twin brothers was analyzed by DNA profiling (DNA Diagnostics Centre Finland).

### Statistics

The *GNRHR* genotype frequency estimates for normal population were calculated from available allele frequency estimates in GnomAD database [19] based on the assumption that Finnish population is in Hardy-Weinberg equilibrium. Since the Phe309del was not listed in GnomAD at all, we used its allele frequency data from our previous work in patients with constitutional delay of growth and puberty [8]. Fisher’s exact test was first used to compare the ratio of each biallelic *GNRHR* mutation (homozygous Arg262Gln and compound heterozygous Gln106Arg/Phe309del) in our series of 9 patients to the estimated genotype frequencies at population level. Finally, we used multinomial probability to estimate the probability to have two patients with rare biallelic *GNRHR* mutations among our series of nine patients. P <0.05 was accepted to indicate statistical significance.

### Ethics

The study had been approved by the Ethics Committee of the Hospital District of Helsinki and Uusimaa. The participants and/or their guardians gave their written informed consents for the genetic studies according to the Declaration of Helsinki.

## Results

### *GNRHR* mutations among adolescent and young adult females with clinical and biochemical signs suggesting partial gonadotropin deficiency

All patients had originally been referred for investigations of partial puberty or secondary amenorrhea at the age of 16.0–18.8 years. The clinical features of these patients are presented in Table 1. In brief, none of them was underweight or persistently obese for their age (patients #8 and #9 had presented with fluctuations in their weight but the weights had been stable and within the normal limits already for few years before being remitted to the tertiary medical center), Patient #5 reported a history of excessive exercise (competitive athlete), however she also had a sister with delayed puberty but no history of excessive exercise. Patient #6 was on L-thyroxine and was biochemically and clinically euthyroid. Thyroid function tests were performed in 8/9 girls, anemia was ruled out in 7/9, and hyperprolactinemia and celiac disease in 5/9. All patients had some degree of breast development before HRT, although Tanner staging was not available for patient #7 (Table 1). However, she had irregular menstruations for three years before secondary amenorrhea at 16 years of age, consistent with partially functioning HPG axis. Patient #8 had oligomenorrhea for three years before secondary amenorrhea at 15 years of age.

**Table 1:** Clinical characteristics and *GNRHR* genotypes in adolescent and young adult females with clinical and biochemical findings suggesting partial gonadotropin deficiency.

Patient	Reason for referral/age	Family historyof delayed puberty	Spontaneous pubertaldevelopment (Tanner stage)	Serum hormone levels^a^			Findings on brain MRI/ pelvic ultrasound	*GNRHR* genotype
				FSH (IU/L)	LH (IU/L)	E2 (nmol/L)		
#1	1°amenorrhea/16.6	No	M4-5P4	1.0–2.5	0.3–2.1	0.019^b^-0.14	normal brain MRI/thin endometrium	c.317A>G p.(Gln106Arg) and c.924_926delCTT p.(Phe309del)
#2	1°amenorrhea/16.0	No	M3	4.7	4.2	0.15	normal brain MRI/thin endometrium	normal
#3	1°amenorrhea/16.5	Yes	M4P3	4.0–5.4	1.9–3.3	<0.02–0.25	small pituitary/prepubertal uterus	normal
#4	1°amenorrhea/17.8	Yes	M3P4	5.0–6.3	3.4–4.6	0.14–0.15	NA/thin endometrium	normal
#5	1°amenorrhea/16.6	Yes	M3P3-4	6.7	1.6	0.12	NA/thin endometrium	normal
#6	1°amenorrhea/18.8	No	M5P4	4.4–5.0	5.6–7.2	0.14–0.32^b^	NA/thin endometrium	normal
#7	2°amenorrhea/18.8	No	Yes^c^	2.2–7.7	1.2–4.7	0.03–0.32	NA/thin endometrium	c.785G>A p.(Arg262Gln)^d^
#8	2°amenorrhea/18.2	No	M5P4-5	0.1–6.0	0.1–1.1	0.04–0.08	normal brain MRI/thin endometrium, adult size uterus	normal
#9	1°amenorrhea/16.5	No	M2P2	0.2–0.3	<0.1	0.02–0.05	normal brain MRI/thin endometrium, small uterus	normal

^a^single measurement or range

^b^measured by mass spectrometry

^c^Tanner staging not available

^d^homozygous mutation

Genetic testing revealed biallelic *GNRHR* mutations in two of nine (22%) (95%CI, 6–55%) female patients (Table 1). No mutations were found in *FGFR1*, *TAC3*, or *TACR3*.

A compound heterozygous c.317A>G p.(Gln106Arg) and c.924_926delCTT p.(Phe309del) mutation in *GNRHR* was found in patient #1. Her healthy parents were heterozygous carriers (mother: c.924_926del CTT p.(Phe309del), father: c.317A>G p.(Gln106Arg)). Patient #1 had thelarche at the age of 11–12 years and spontaneous breast development up to Tanner stage M4-5. However, she had primary amenorrhea, and was referred to a tertiary center at the age of 16.6 years. The laboratory measurements at the age of 16.6–17.2 years (before estrogen treatment) showed constantly low estradiol (0.02 nmol/L), FSH 1.0–1.3 IU/L and LH 0.3–0.7 IU/L levels. In GnRH stimulation test, at the age of 16.7 years, the peak FSH (3.1 IU/L) and LH (5.9 IU/L) levels were indistinguishable from levels seen in patients with constitutional delay of growth and puberty. At the age of 17.9 years, she initiated hormone replacement therapy (HRT), which induced menarche at the age of 19.1 years. At the age of 19.5 years the HRT was ceased, and, when measured four months later, her estradiol level was low (0.019 nmol/L), although her basal FSH (2.4 IU/L) and LH (1.7 IU/L) levels were normal, and GnRH-induced peak FSH (5.5 IU/L) and LH (18.1 IU/L) levels were pubertal. She had no spontaneous menstrual bleeding and ultrasound examination showed that the uterus had diminished in size. These findings were consistent with partial gonadotropin deficiency.

Patient #7 was found to harbor a homozygous c.785G>A p.(Arg262Gln) mutation in exon 3 of *GNRHR*. Since the parents’ DNA samples were not available, we verified the normal copy number of *GNRHR* exon 3 with qPCR analysis. This patient had spontaneous menarche at the age of 13 years, three years of irregular spontaneous menstruations and secondary amenorrhea at the age of 16.0 years. She was treated with oral contraceptives and HRT (latter from 19.7 years of age onwards). The HRT was discontinued at the age of 25.0 years. Thereafter she had a single uterine bleeding. After one month of HRT cessation, the laboratory values of gonadotropin and sex hormones were normal: estradiol 0.32 nmol/L, FSH 3.9 IU/L, LH 3.4 IU/L, and AMH 1.0 ug/L. However, with further HRT withdrawal, secondary amennorhea occurred, and within six months she had slipped back to hypogonadal state. The ovarian ultrasound examination revealed low antral follicle count (3/0) which suited with the low AMH level (1.2 ug/L). Moreover, her serum estradiol was undetectably low (< 0.07 nmol/L) without elevated basal gonadotropin levels (FSH 4.5 IU/L and LH 3.2 IU/L). All these findings were consistent with partial gonadotropin deficiency.

### Enrichment of biallelic *GNRHR* mutations among adolescents and young adults presenting with signs of partial gonadotropin deficiency

We estimated the genotype frequencies for the homozygous Arg262Gln genotype and Gln106Arg/Phe309del compound heterozygous genotype in the Finnish population by using the allele frequencies available from the GnomAD database [19] and our previous work [8]. The respective estimated genotype frequencies at population level are 1:35426, and 1:75335. The occurrence of each of these genotypes alone in our series of 9 patients indicated significant enrichment as compared to population level estimates (P<0.0005 and P<0.0002, respectively). The probability to have these two patients with biallelic *GNRHR* mutations among our series of nine patients was estimated to be very low, approximately one to thirty-seven million.

### Monozygotic twin brothers with stalled puberty and biallelic *GNRHR* mutations

**Fig 1:**
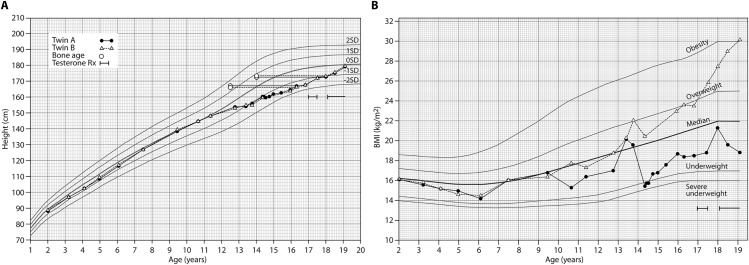
Growth charts of monozygotic twin brothers who presented with stalled puberty due to biallelic mutations in *GNRHR* (c.317A>G p.(Gln106Arg) and c.785G>A p.(Arg262Gln)). A. Longitudinal growth of the brothers was practically identical. Note the absence of pubertal growth spurt. B. The brothers were discordant for weight. The reference ranges for body mass index (BMI) were modified from [20].

The brothers were referred to the Helsinki University Hospital Paediatric Endocrine outpatient clinic for the assessment of puberty. They had no history of cryptorchidism or micropenis, and their developmental milestones had been normal; nor were there signs of chronic illnesses underlying their pubertal delay. They continued to grow with prepubertal growth velocity (Fig 1A). Interestingly, their weights had started to deviate from 9.5 years (Fig 1B); at the age of 14.5 years, twin A had been investigated for weight loss, but no underlying cause had been identified. Since then, his BMI-for-age had remained relatively stable, whereas his twin brother had continued to gain weight (Fig 1B).

The clinical and biochemical data of the twins are presented in Table 2. In brief, based on the Tanner stage G2P2 and pubertal levels of LH, FSH and inhibin-B at the age of 16.3, the boys were considered to have constitutional delay of growth and puberty, and watchful waiting was commenced (Table 2). Due to poor spontaneous progression of puberty, however, GnRH stimulation test was performed, and it revealed pubertal LH responses. Brain MRI scans were normal, although Twin B had an incidental arachnoid cyst. A low-dose monthly testosterone treatment increased height velocities in both boys (Fig 1A). Twin B, heavier of the boys, had consistently lower serum testosterone levels (Table 2).

**Table 2:** Clinical and hormonal findings in two monozygotic twin brothers who presented with stalled puberty due to compound heterozygous mutations in the *GNRHR* gene (c.317A>G p.(Gln106Arg) and c.785G>A p.(Arg262Gln)).

Age (years)	Testis size (mL)	Tanner stage	LH (IU/L)	FSH (IU/L)	Testosterone (nmol/L)	Inhibin B (ng/L)
16.3						
Twin A	3.2	G2P2	2.3	2.6	1.7	119
Twin B	3.2	G2P2	1.9	1.8	0.7	103
16.8						
Twin A	4.5	G2P2	2.3 (19.7/60’)^a^	2.3 (5.1/20’,30’)^a^	2.1	122
Twin B	3.6	G2P2	1.7 (19.3/60’)	1.9 (4.5/30’)	0.9	131
17.5^b^						
Twin A	5.2	G3P3	1.9	2.4	4.2	NA
Twin B	4.9	G3P3	1.8	1.8	3.7	NA
18.0						
Twin A	4.0	G3-4P3	1.6	2.0	2.4	NA
Twin B	4.5	G3P3	1.6	1.7	1.3	NA
18.5^c^						
Twin A	5.0	G4P4	NA	NA	NA	NA
Twin B	5.0	G4P4	NA	NA	NA	NA
19.1^c^						
Twin A	7.0	G5P5	3.4	6.8	21.5	88
Twin B	6.6	G4P4	2.8	4.0	7.8	88

As shown in Fig 1, the Twin B was heavier than his brother.

^a^The values in parentheses are peak values / timings of peak values in the GnRH stimulation test

^b^The values at 17.5 years of age are measured after 6 months of low-dose testosterone therapy

^c^The boys received continuous testosterone therapy for hypogonadotropic hypogonadism from the age of 18.1 years onwards

Genetic testing was performed at the age of 18.1 years. Both brothers harbored compound heterozygous mutations in *GNRHR*; c.317A>G p.(Gln106Arg) and c.785G>A p.(Arg262Gln), and their healthy parents were heterozygous carriers (mother: c.785G>A p.(Arg262Gln), father: c.317A>G p.(Gln106Arg)). Monozygosity of the twins was confirmed with DNA profiling.

The boys were commenced on testosterone treatment for CHH at the age of 18.1 years. At the age of 19.1 years, laboratory data (27 days after previous exogenous testosterone (Sustanon) injection), together with the enlarged testicular volume (from 4 mL to 7 mL), suggested reversal of CHH in Twin A, who at this point had serum T and gonadotropin levels within adult reference range (Table 2). Also Twin B showed some testicular growth and rise in serum T and gonadotropin levels, indicative of partially functioning HPG-axis. However, his testosterone level was still clearly lower than his brother’s (Table 2).

Serum transaminases and lipid levels were measured in the clinical laboratory at the age of 18.8 years and they were within the normal range in both patients; HDL-cholesterol level, however, was slightly lower in twin B (1.72 mmol/L vs. 1.31 mmol/l in Twin A vs. Twin B). The SHBG levels of the boys were measured at the age of 19.1 years and they were 32 nmol/l (Twin A) and 24 nmol/l (Twin B). Using the Vermeulen formula [21], the calculated free testosterone levels were 0,479 nmol/l / 2.23% in Twin A; and 0.179 nmol/l / 2.29% in Twin B.

## Discussion

We examined adolescents and young adults with stalled puberty or unexplained amenorrhea for mutations in *GNRHR*, *FGFR1*, *TAC3*, and *TACR3* genes. This study was sparked by previous works, which have demonstrated a wide phenotypic spectrum in females with CHH and a putative role of CHH genes in the etiology of hypothalamic amenorrhea [4,9,12]. Herein, we hypothesized that biallelic *GNRHR* mutations could be enriched among adolescent and young adult females presenting with clinical and biochemical signs suggesting partial gonadotropin deficiency, and found statistical evidence to support this notion, since two patients in our series of nine harbored biallelic mutations in *GNRHR*. The patient harboring a compound heterozygous *GNRHR* mutation p.(Gln106Arg) and p.(Phe309del) had spontaneous breast development, primary amenorrhea, low estradiol levels with low-normal gonadotropin levels and normal pubertal LH response to GnRH stimulation. Her clinical presentation was in accordance with a study showing that the p.(Gln106Arg) mutation decreases the binding of GnRH on the receptor causing a partial loss-of-function [10]. Previous clinical evidence also link this mutation to partial CHH: the p.(Gln106Arg) mutation has been described in a homozygous state in a female patient with partial CHH [22], and in compound heterozygous state in female patients with partial puberty [10,23]. Pitteloud *et al*. [24] have described a male patient homozygous for the p.(Gln106Arg) mutation in *GNRHR* with a partial phenotype who underwent reversal of hypogonadotropic hypogonadism, and a partial CHH phenotype was described in a Brazilian male patient homozygous for this mutation [9]. Very recently the phenotypic spectrum associated to p.(Gln106Arg) mutation was expanded to polycystic ovarian syndrome [25]. The p.(Phe309del) mutation has been described in a compound heterozygous state together with a p.(Arg262Gln) mutation in a CHH reversal patient [26]. Our patient with the homozygous p.(Arg262Gln) mutation presented with secondary amenorrhea associated with normal gonadotropin levels, and probably normal pubertal development. The p.(Arg262Gln) mutation has previously been shown to impair signal transmission and cause a partial loss-of-function of the GnRH receptor [10]. Accordingly, previously described patients carrying homozygotic p.(Arg262Gln) mutations have partial CHH phenotypes [13,27], and we have previously reported two Finnish patients with reversal of CHH carrying p.(Arg262Gln) mutation in a compound heterozygous state [26]. Interestingly, Caronia *et al*. [4] found a heterozygous p.(Arg262Gln) *GNRHR* mutation in a patient with hypothalamic amenorrhea. Taken together, phenotypic variability is an important theme among *GNRHR* mutation carriers [11,14,28,29], and it would be important to replicate our results in other populations with relatively high carrier frequency of *GNRHR* mutations [30]. In general, the partial puberty phenotypes are problematic, because such patients might not be detected timely; the Finnish puberty screen for example relies first on the development of breast tissue before the age of 13 years [31]. Indeed, slowly progressing puberty with primary amenorrhea may in ambiguous cases be unnecessarily attributed to eating disorders or excessive physical training, and the onset of sex hormone replacement is remarkably delayed. Importantly, the diagnosis of functional hypothalamic amenorrhea should only be made after exclusion of pathologies [32,33]. It may be argued that our two female patients with biallelic *GNRHR* mutations, to some extent, resemble adult onset hypogonadotropic hypogonadism. To the best of our knowledge, there are no systematic descriptions of females with this condition, contrary to its well-known concept in males [34–36]. It should be noted that women with normosmic CHH, an underinvestigated topic, are diagnosed on average about nine years later than our patients [12].

Our study sheds light on environmental factors in determining the CHH phenotype in males. We describe the first monozygotic twin brothers carrying compound heterozygous *GNRHR* mutations p.(Gln106Arg) and p.(Arg262Gln) who presented with stalled puberty, and were concordant for height but discordant for weight, which is quite unusual for monozygotic twins [37]. The twin with the higher BMI-for-age had constantly ~50% lower spontaneous serum T levels than his brother, suggesting modification of the HPG axis by adipose tissue. Alternative explanations for the difference in sex steroid levels appear unlikely, as the patients have lived together throughout their lives, had similar birth weights, and share the fundamental genetic and environmental factors potentially affecting the timing of puberty. At 19 years of age, the boys were on testosterone treatment, and the difference in their weight and spontaneous serum T levels was further pronounced. Interestingly, the twin with the lower BMI-for-age showed signs of reversal of CHH. It is tempting to speculate that changes in the metabolic status of a patient with partial CHH contributes to the reversal of CHH, a phenomenon frequently reported also in patients carrying biallelic *GNRHR* mutations [22,24,26,34]. Indeed, obesity during adolescence modifies HPG-axis activity in boys without reproductive disorders by increasing aromatization of androgens to estrogens and circulating estradiol, which is a well-established negative regulator of gonadotropin secretion in pubertal boys [38–40]. This obesity-related suppression of gonadotropin secretion provides a plausible explanation to observed difference in testosterone levels, particularly as also free testosterone was lower in the overweight twin.

We conclude that biallelic mutations in *GNRHR* are enriched among adolescents and young adults presenting with clinical and biochemical signs of partial gonadotropin deficiency, and our results suggest that genetic testing of *GNRHR* should be offered to adolescent females with low-normal gonadotropins and unexplained stalled puberty or menstrual dysfunction. In males, excess adipose tissue may suppress HPG axis in patients with partial form of gonadotropin deficiency.

## Supporting information

S1 Table Primer sequences used in molecular genetic analyses. (XLSX)
